# Hypothermia increases adenosine monophosphate and xanthosine monophosphate levels in the mouse hippocampus, preventing their reduction by global cerebral ischemia

**DOI:** 10.1038/s41598-024-53530-1

**Published:** 2024-02-07

**Authors:** Masaru Doshi, Yujin Natori, Akira Ishii, Daisuke Saigusa, Shiro Watanabe, Makoto Hosoyamada, Yutaka Hirashima-Akae

**Affiliations:** 1 Department of Human Physiology and Pathology, Faculty of Pharma-Sciences, Teikyo University, 2-11-1 Kaga, Itabashi-ku, Tokyo, 173-8605 Japan; 2 Department of Legal Medicine and Bioethics, Nagoya University Graduate School of Medicine, 65 Tsurumai-cho, Showa-ku, Nagoya, 466-8550 Japan; 3 Department of Biomedical and Analytical Sciences, Faculty of Pharma-Sciences, Teikyo University, 2-11-1 Kaga, Itabashi-ku, Tokyo, 173-8605 Japan; 4 Division of Nutritional Biochemistry, Institute of Natural Medicine, University of Toyama, 2630 Sugitani, Toyama, 930-0194 Japan; 5 Akae Clinic, 310 Horioka, Imizu, Toyama 933-0223 Japan

**Keywords:** Metabolomics, Cell death in the nervous system, Neurological disorders, Experimental models of disease

## Abstract

Global cerebral ischemia (GCI) caused by clinical conditions such as cardiac arrest leads to delayed neuronal death in the hippocampus, resulting in physical and mental disability. However, the mechanism of delayed neuronal death following GCI remains unclear. To elucidate the mechanism, we performed a metabolome analysis using a mouse model in which hypothermia (HT) during GCI, which was induced by the transient occlusion of the bilateral common carotid arteries, markedly suppressed the development of delayed neuronal death in the hippocampus after reperfusion. Fifteen metabolites whose levels were significantly changed by GCI and 12 metabolites whose levels were significantly changed by HT were identified. Furthermore, the metabolites common for both changes were narrowed down to two, adenosine monophosphate (AMP) and xanthosine monophosphate (XMP). The levels of both AMP and XMP were found to be decreased by GCI, but increased by HT, thereby preventing their decrease. In contrast, the levels of adenosine, inosine, hypoxanthine, xanthine, and guanosine, the downstream metabolites of AMP and XMP, were increased by GCI, but were not affected by HT. Our results may provide a clue to understanding the mechanism by which HT during GCI suppresses the development of delayed neuronal death in the hippocampus.

## Introduction

Global cerebral ischemia (GCI) is induced when cardiac arrest occurs for any reason, and if blood flow is not resumed as quickly as possible, neurons in the brain will die and severe sequelae are likely to result even if the patient is resuscitated^1,2^. This clinical condition is known as post-cardiac arrest syndrome, and its prognosis is extremely poor. Therapeutic hypothermia (HT) is one of the most effective treatments to alleviate the sequelae^3^. However, the effectiveness of HT itself varies from case to case, and there are many issues that need to be resolved to establish standard protocols such as cooling methods, duration, and temperature^4^. To solve these problems, it is necessary to elucidate the pathophysiology of GCI and the molecular mechanism of the neuroprotective effect of HT on it.

There are two types of experimental model of cerebral ischemia: the middle cerebral artery occlusion (MCAO) model and the bilateral common carotid artery occlusion (BCCAO) model in mice and rats. The former is a model of cerebral infarction in which a cerebral artery is locally occluded, and the latter is a model of cardiac arrest in which the occlusion of the left and right common carotid arteries temporarily reduces the blood flow to the entire brain. Transient GCI caused by BCCAO induces delayed neuronal death in the hippocampus^5^. Although many investigators have intensively studied the pathogenesis after GCI–reperfusion^6–8^, the mechanism of delayed neuronal death in the hippocampus after GCI has not yet been clearly elucidated. Previously, using the BCCAO model, we showed that HT during GCI did not cause delayed neuronal death in the hippocampus^9^. The BCCAO model is characterized by a considerable time lag between ischemic treatment and the onset of delayed neuronal death in the hippocampus so that neuronal death has not yet occurred at the end of GCI treatment. Since a very marked difference in the occurrence of delayed neuronal death in the hippocampus was observed only at different body temperatures during GCI under our experimental conditions, we considered that clarifying the molecular changes in the hippocampus caused by this difference in body temperature leads to the understanding of the mechanism by which HT inhibits the occurrence of delayed neuronal death in the hippocampus after reperfusion. The finding in our previous study that HT during GCI inhibits the development of delayed neuronal death in the hippocampus was demonstrated by histological evaluation^9^. However, a quantitative evaluation system is required to elucidate the mechanism of delayed neuronal death in the hippocampus.

In recent years, omics technology has been developed to comprehensively analyze various molecules such as genomes, transcripts, proteins, and metabolites of lipids and carbohydrates in vivo. Compared with genome and proteome analyses, metabolome analysis has the advantage of providing information closer to phenotypes and has led to the discovery of biomarkers for various diseases and the elucidation of their pathophysiological mechanisms^10,11^. Several studies have been conducted using the latest metabolomic techniques to investigate changes in the levels of brain metabolites in the MCAO model^12,13^, providing useful information for understanding the pathophysiology of cerebral infarction. In addition, Rashad et al. have recently investigated in detail the changes in levels of metabolites in the hippocampus after reperfusion using a rat model of GCI and found that pyrimidine and purine metabolisms were changed^14^. However, there has been no study in which changes in the levels of metabolites in the hippocampus due to HT during GCI were investigated.

In this study, to evaluate in detail the effect of HT during GCI on the development of delayed neuronal death in the hippocampus, we first examined the occurrence of delayed neuronal death in the hippocampus after GCI–reperfusion over time using an evaluation system that quantifies nucleosomes, which are DNA fragments formed after cell death, as previously reported^15^. We then compared metabolic changes in the hippocampus at 37 °C (normothermia, NT) with those at 34 °C (HT) during GCI by metabolome analysis to clarify the mechanism by which HT during GCI prevents the development of delayed neuronal death in the hippocampus.

## Results

### Inhibition of the development of delayed neuronal death in hippocampus by HT during GCI

**Figure 1:**
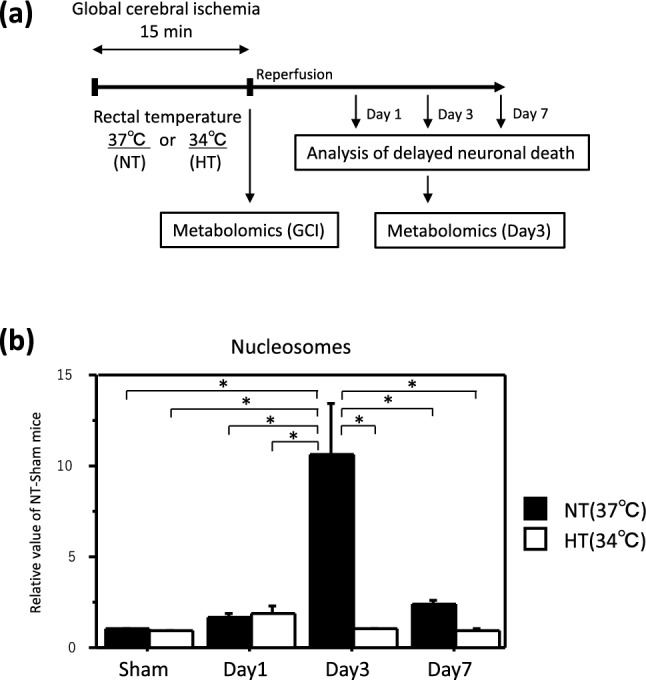
HT during GCI suppressed the development of delayed neuronal death in the hippocampus. Experimental schedule (**a**). GCI was induced by BCCAO for 15 min under 1.5% isoflurane anesthesia in both NT and HT groups. Nucleosome levels in the hippocampus 1, 3, and 7 days after reperfusion (**b**) were determined as described in Materials and Methods. Control mice (Sham) were sham-operated without BCCAO under isoflurane anesthesia. Data are expressed as the mean and standard error of the mean for the six male mice. Statistical analyses were performed by two-way ANOVA followed by Bonferroni/Dunn post hoc test (*p < 0.0018).

We evaluated the occurrence of delayed neuronal death in the hippocampus after GCI–reperfusion according to the experimental schedule shown in Fig. 1a. The nucleosome level after GCI–reperfusion in the NT group was significantly increased 3 days after reperfusion compared with the sham-operated mice (Fig. 1b). On the other hand, the nucleosome level after GCI–reperfusion in the HT group was not significantly different from that in the sham-operated mice on any day after reperfusion and was significantly lower than that in the NT group after 3 days of reperfusion. These results indicate that HT during GCI inhibits the progression to delayed neuronal death in the hippocampus.

### Metabolomics after GCI

**Figure 2:**
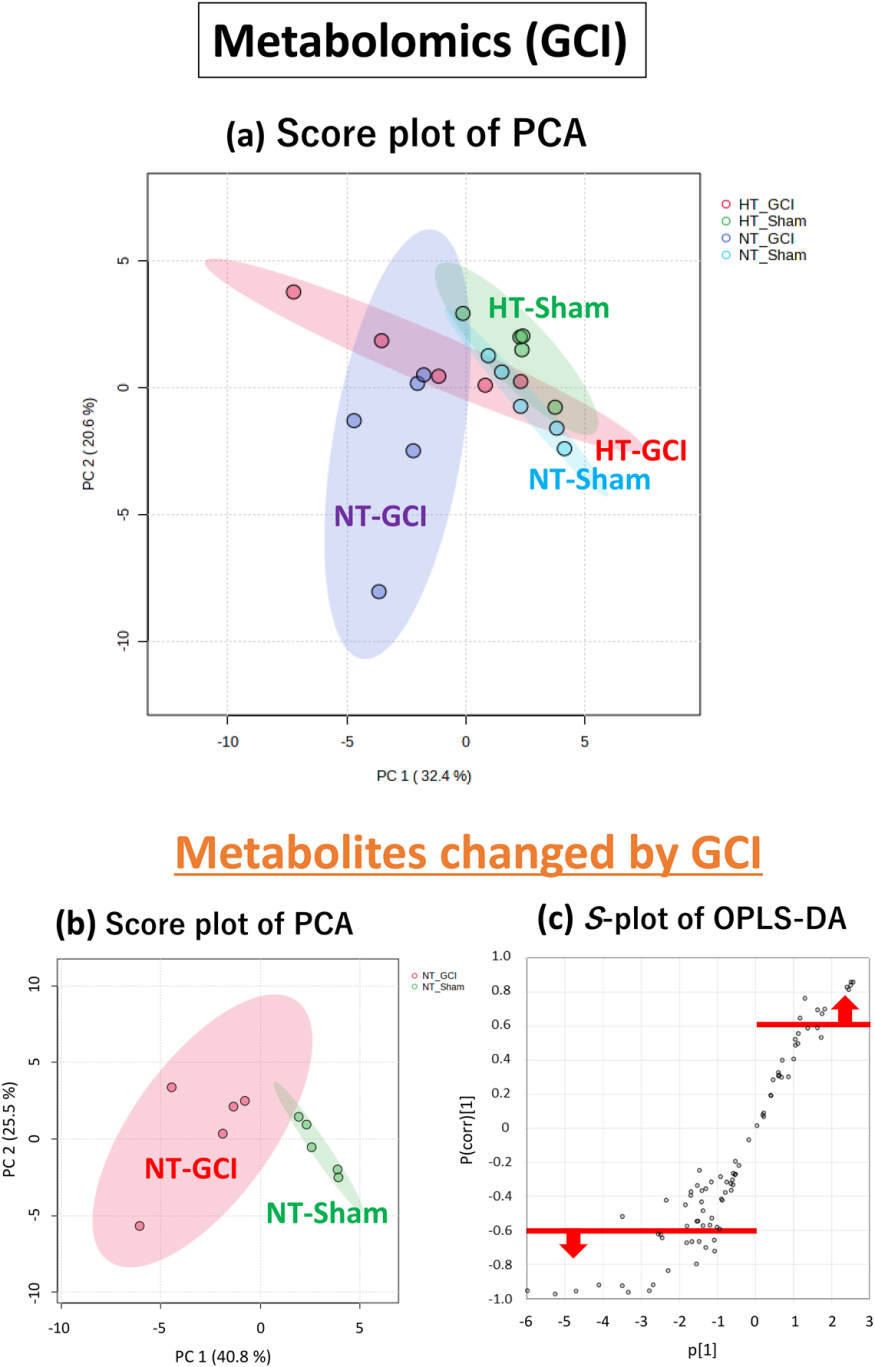

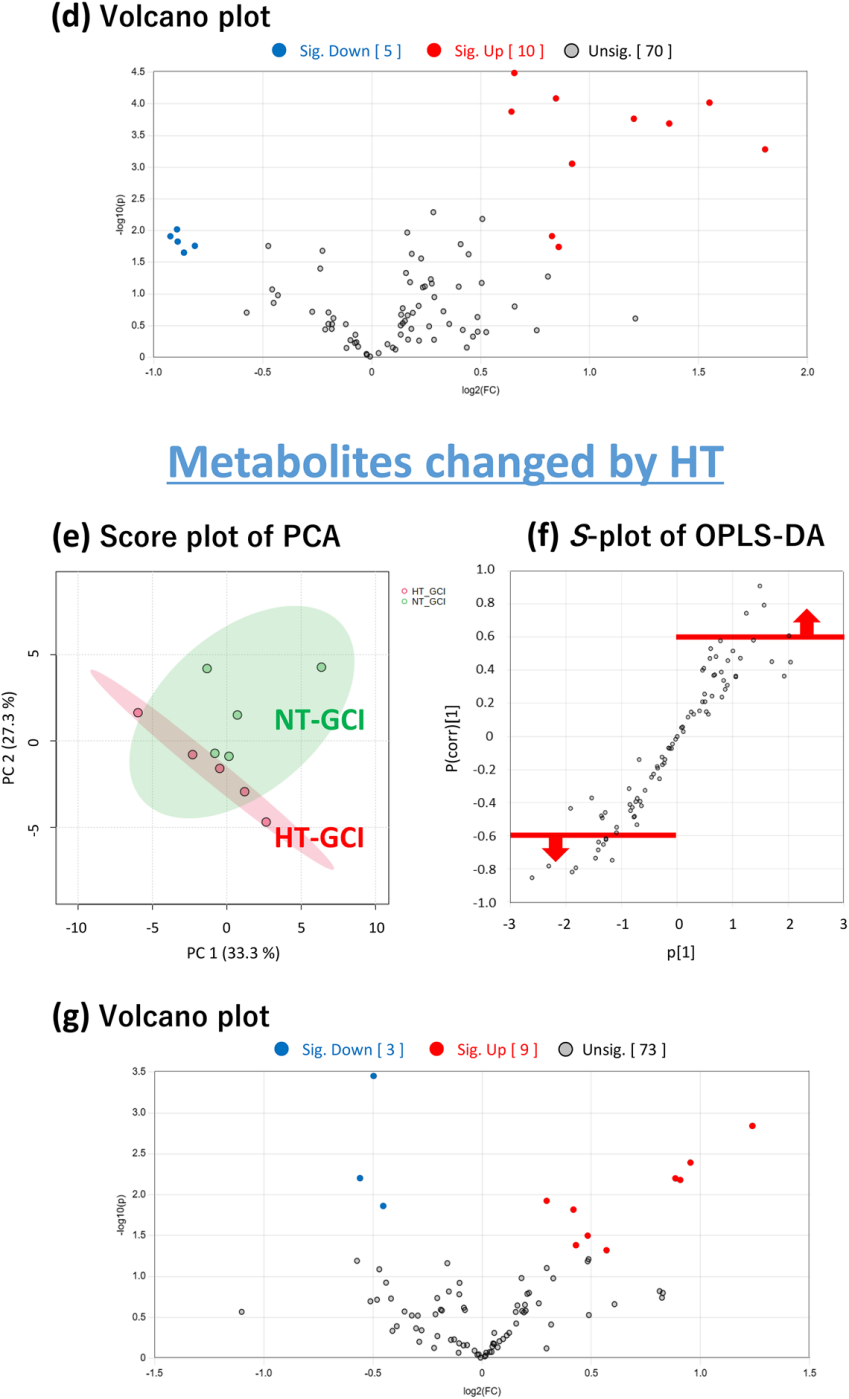
Multivariate analysis of the hippocampus immediately after the GCI. Score plot of PCA of all groups (**a**). Score plot of PCA of the sham-operated and GCI mice of NT group. The green and red circles indicate sham-operated and GCI mice of NT groups, respectively (**b**). *S*-plot of OPLS-DA. The vertical axis is the correlation coefficients (p (corr)^1^): the farther up or down of the axis, the more significant the difference. The horizontal axis is the covariance (p^1^): the farther to the left or right of the axis, the greater the change in level. As shown by the red line, metabolites with correlation coefficients above 0.6 are those whose levels were decreased by GCI and metabolites with correlation coefficients below − 0.6 are those whose levels were increased by GCI: these metabolites were selected (**c**). Volcano plot. The vertical axis is the − log_10_ [*p*-value], with a larger value indicating a more significant difference. The horizontal axis is the log_2_ [fold change], indicating that the farther to the left or right of the axis, the greater the amount of change. Blue circles indicate the metabolites whose levels were significantly decreased by GCI and red circles indicate those whose levels were significantly increased by GCI (**d**). Score plot of PCA. The green and red circles indicate NT and HT groups of GCI mice, respectively (**e**). As shown by the red line, metabolites with correlation coefficients above 0.6 are those whose levels were decreased by HT and metabolites with correlation coefficients below − 0.6 are those whose levels were increased by HT: these metabolites were selected (**f**). Volcano plot. Blue circles indicate the metabolites whose levels were significantly decreased by HT and red circles indicate those whose levels were significantly increased by HT (**g**).

Initially, we performed a metabolome analysis focusing on the time point immediately after the GCI, which reflects metabolite changes during GCI for 15 min. PCA score plots for all groups after GCI for 15 min are shown in Fig. 2a. There was a tendency for metabolites to separate on the score plots between sham-operated and GCI mice in both NT and HT groups. There was also a tendency for metabolites to separate on the score plot between the NT and HT groups in both sham-operated and GCI mice. Therefore, the metabolites in the hippocampus were found to be changed by GCI or HT, respectively.

### Metabolites changed by GCI

**Figure 3:**
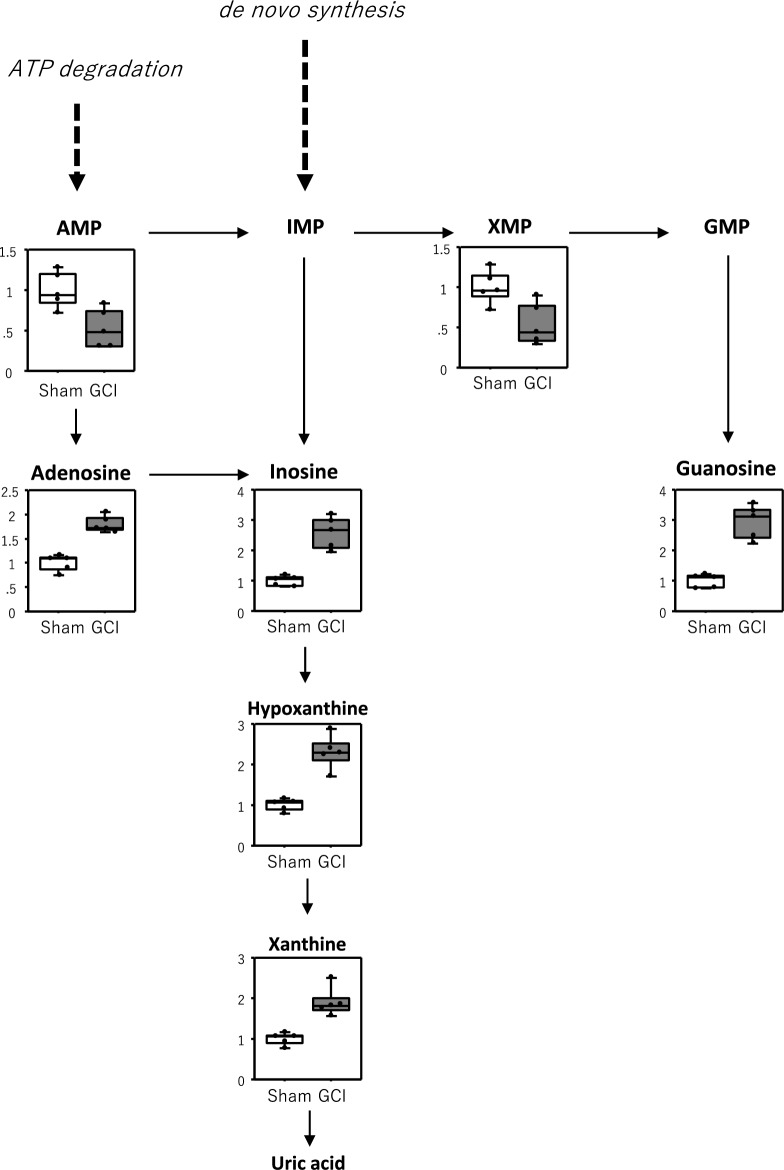
Purine metabolic pathway changed by GCI. Seven of the 13 metabolites listed in Table 1 were purine metabolites. These are shown in the graphs, respectively. Values are shown relative to the sham-operated mice (Sham).

Next, to identify metabolites that showed changes in their levels in the hippocampus after GCI, we performed metabolome analysis of the hippocampus of the GCI and sham-operated mice of the NT group. Principal component analysis (PCA) of the 85 metabolites quantified in the hippocampus of the GCI and sham-operated mice showed a tendency for the metabolites to separate on the score plot (Fig. 2b). Orthogonal partial least squares-discriminant analysis (OPLS-DA) was then performed to determine which of these metabolites were responsible for separating these groups. Metabolites with correlation coefficients above 0.6 and below − 0.6 in the *S*-plot (Fig. 2c) were selected, and from those metabolites, 15 metabolites with significant differences narrowed down in the Volcano plot (Fig. 2d) are listed in Table 1. Eleven of these metabolites are involved in nucleic acid metabolism and seven are involved in purine metabolism, suggesting that the purine metabolic pathway was mainly changed (Fig. 3).

**Table 1:** List of metabolites changed by GCI.

Compound	Fold change (FC)	Log_2_(FC)	VIP	− log_10_(p)	Raw *p*-value	FDR
**Metabolomics (GCI)**						
2-Aminoethanol	1.57	0.65	1.68	4.49	0.00003	0.0027
Adenosine	1.80	0.84	1.70	4.09	0.00008	0.0027
Guanosine	2.93	1.55	1.72	4.02	0.0001	0.0027
Uracil	1.56	0.64	1.62	3.88	0.0001	0.0028
Hypoxanthine	2.30	1.20	1.62	3.76	0.0002	0.0029
Inosine	2.58	1.37	1.69	3.69	0.0002	0.0029
Ribose	3.49	1.80	1.68	3.28	0.0005	0.0064
Xanthine	1.89	0.92	1.63	3.06	0.0009	0.0094
Sorbose	0.54	− 0.89	1.52	2.02	0.0096	0.0741
Adenosine monophosphate	0.53	− 0.92	1.51	1.91	0.0123	0.0744
Uridine	1.77	0.83	1.14	1.91	0.0123	0.0744
Xanthosine monophosphate	0.54	− 0.89	1.48	1.83	0.0149	0.0810
Psicose	0.57	− 0.81	1.46	1.76	0.0174	0.0810
2′-Deoxyuridine	1.81	0.86	1.10	1.74	0.0181	0.0810
Tagatose	0.55	− 0.86	1.44	1.65	0.0222	0.0872
**Metabolomics (Day 3)**						
Adenosine	0.44	− 1.18	1.88	3.57	0.0003	0.0096
2-Aminoethanol	1.57	0.65	1.87	3.53	0.0003	0.0096
Methylsuccinic acid	1.63	0.71	1.84	3.17	0.0007	0.0164
2-Hydroxyisovaleric acid	1.90	0.93	1.75	2.77	0.0017	0.0260
5-Methoxytryptamine	2.23	1.15	1.76	2.72	0.0019	0.0260
Putrescine	8.18	3.03	1.71	2.67	0.0021	0.0260
Cycteine	1.52	0.60	1.63	2.27	0.0054	0.0478
Glucose	2.76	1.47	1.55	1.94	0.0115	0.0901
Erythrulose	0.55	− 0.86	1.53	1.83	0.0149	0.0977
3-Hydroxypyruvic acid	0.52	− 0.96	1.37	1.43	0.0375	0.1672
Hypoxanthine	1.67	0.74	1.32	1.35	0.0444	0.1892

### Metabolites changed by HT

To identify metabolites that were changed by HT during GCI, metabolome analysis of the hippocampus of the NT and HT groups of GCI mice was performed. PCA of the NT and HT groups showed a tendency for the metabolites to separate on the score plot (Fig. 2e). OPLS-DA was also performed, and metabolites with correlation coefficients above 0.6 and below − 0.6 in the *S*-plot (Fig. 2f) were selected, and from those metabolites, 12 metabolites with significant differences narrowed down in the Volcano plot (Fig. 2g) are listed in Table 2. These metabolites included those involved in metabolic pathways changed only by HT, independent of the metabolic pathways changed by GCI.

**Table 2:** List of metabolites changed by HT.

Compound	Fold change (FC)	Log_2_(FC)	VIP	− log_10_(p)	Raw *p*-value	FDR
**Metabolomics (GCI)**						
Octadecanol	0.71	− 0.50	2.05	3.45	0.0004	0.0301
3-Phosphoglyceric acid	2.36	1.24	1.93	2.84	0.0014	0.0613
Adenosine monophosphate	1.94	0.95	1.85	2.39	0.0040	0.0937
Cysteine	0.68	− 0.56	1.79	2.20	0.0063	0.0937
Creatinine	1.85	0.89	1.77	2.20	0.0064	0.0937
Xanthosine monophosphate	1.88	0.91	1.79	2.18	0.0066	0.0937
Pantothenic acid	1.23	0.30	1.69	1.92	0.0119	0.1443
1,6-Anhydroglucose	0.73	− 0.45	1.68	1.86	0.0138	0.1443
2-Ketoadipic acid	1.34	0.42	1.66	1.82	0.0153	0.1443
Norepinephrine	1.40	0.48	1.55	1.50	0.0318	0.2707
O-Phosphoethanolamine	1.35	0.43	1.47	1.38	0.0413	0.3194
5-Methoxytryptamine	1.49	0.57	1.44	1.32	0.0480	0.3402
**Metabolomics (Day 3)**						
Adenosine	2.02	1.01	2.06	2.51	0.0031	0.2228
2-Deoxy-glucose	3.41	1.77	1.87	2.34	0.0045	0.2228
2-Hydroxyisovaleric acid	0.69	− 0.53	1.69	1.67	0.0216	0.5145
Adenine	1.57	0.65	1.85	1.60	0.0250	0.5145
2-Hydroxyglutaric acid	1.36	0.45	1.65	1.44	0.0360	0.5145
Octadecanol	1.34	0.43	1.49	1.33	0.0466	0.5145

### Metabolites changed by GCI and affected by HT

**Figure 4:**
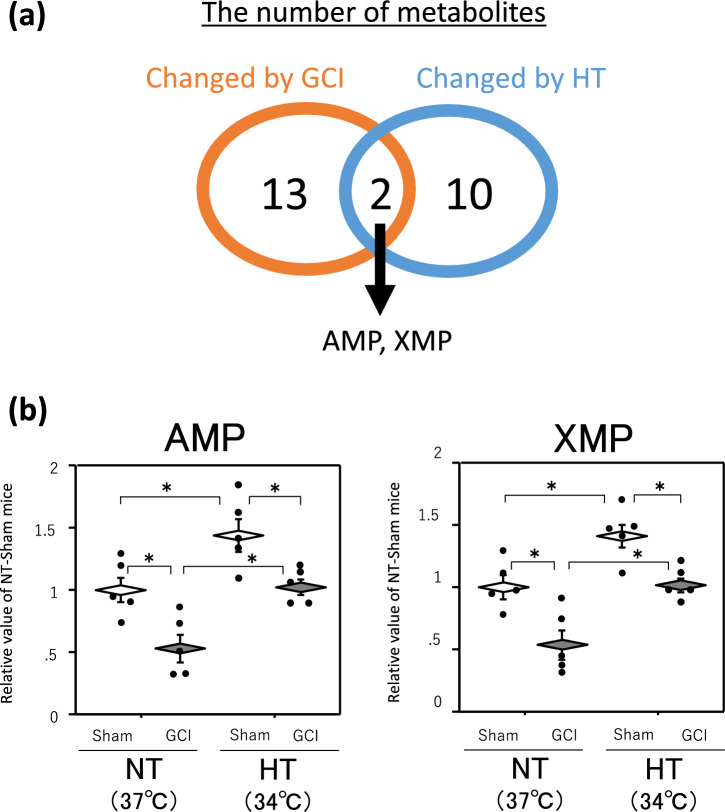

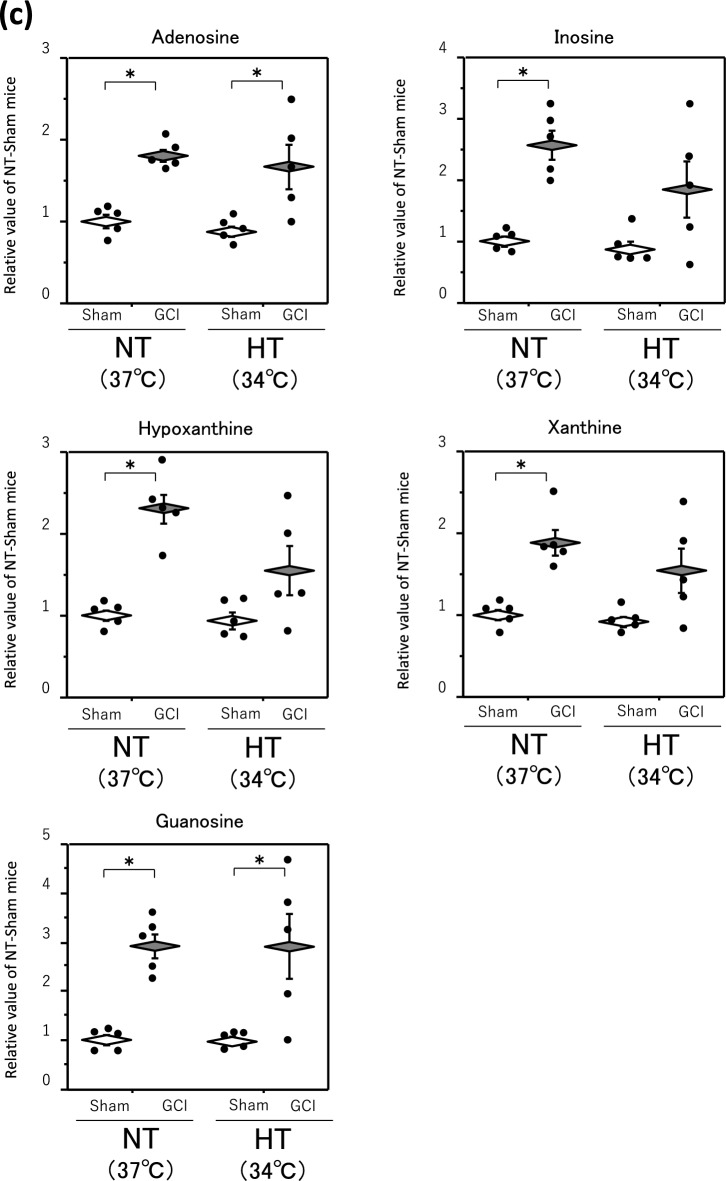
AMP, XMP and other purine metabolites levels in hippocampus. GCI was induced by BCCAO for 15 min under 1.5% isoflurane anesthesia in both NT and HT groups. Each control mouse (Sham) was sham-operated without BCCAO under isoflurane anesthesia. The hippocampus was removed immediately after GCI and metabolome analysis was performed as described in Materials and Methods. Venn diagram showing two shared metabolites from among metabolites whose levels were changed by GCI and HT (**a**). AMP and XMP levels in the hippocampus (**b**). Adenosine, inosine, hypoxanthine, xanthine, and guanosine levels in the hippocampus (**c**). Data are expressed as the mean and standard error of the mean for the five male mice. Statistical analyses were performed by two-way ANOVA followed by Bonferroni/Dunn post hoc test (*p < 0.0125).

Finally, to identify the metabolites changed during GCI and affected by HT, we searched for metabolites found in both Tables 1 and 2 and were able to narrow down to two metabolites, adenosine monophosphate (AMP) and xanthosine monophosphate (XMP) (Fig. 4a). A detailed comparison of these two metabolites is shown in Fig. 4b.

Both AMP and XMP levels in the NT and HT groups were significantly decreased by GCI. In contrast, the AMP and XMP levels in both sham-operated and GCI mice of the HT group were significantly higher than those of the NT group, so that those levels in the GCI mice of the HT group only returned to the levels in the sham-operated mice of the NT group. The levels of adenosine, inosine, hypoxanthine, xanthine, and guanosine, the downstream metabolites of AMP and XMP, in the NT group were significantly higher in GCI mice than in sham-operated mice, but they were not significantly different between the NT and HT groups in either GCI or sham-operated mice (Fig. 4c).

### Metabolomics 3 days after reperfusion

**Figure 5:**
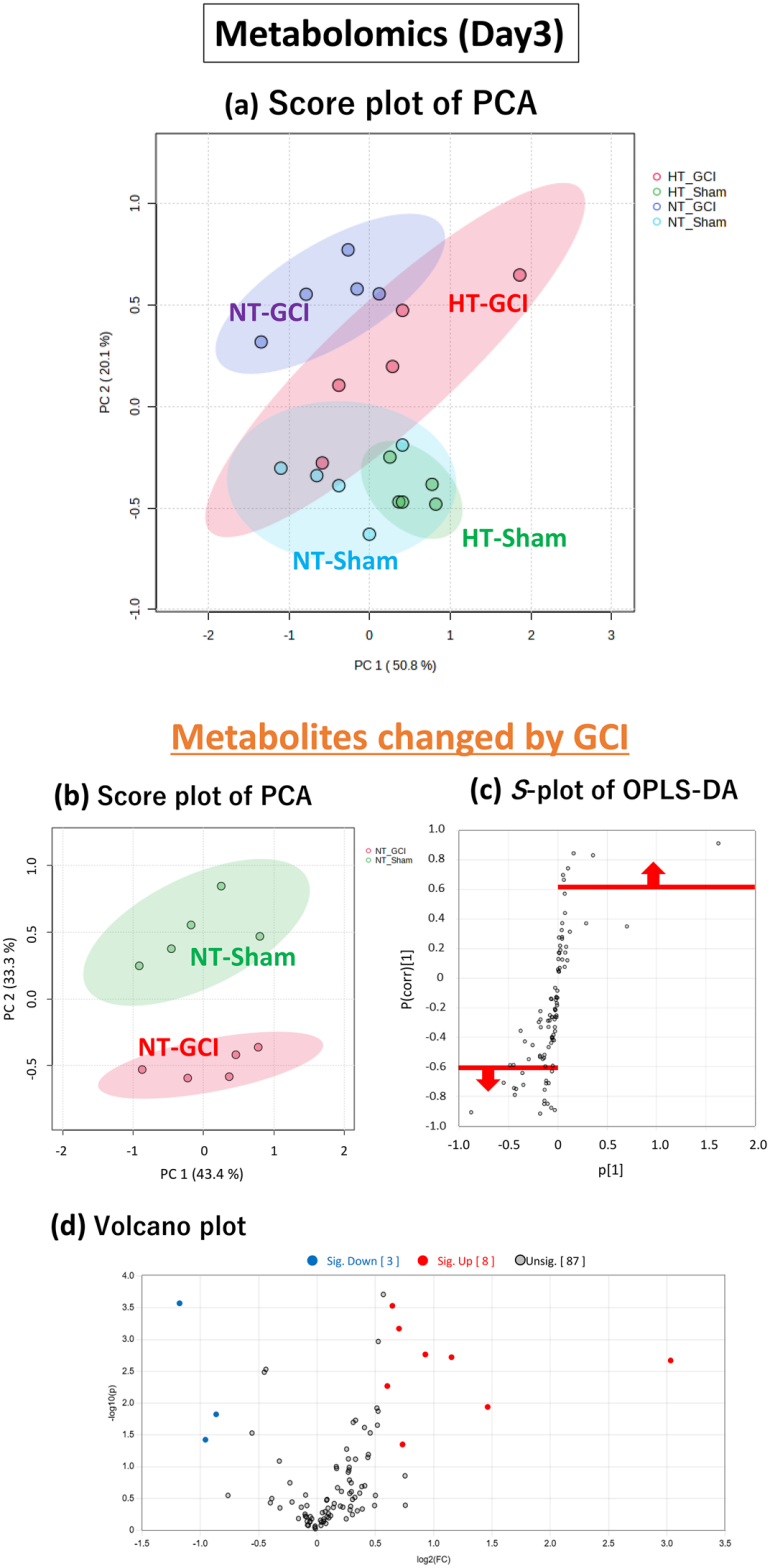

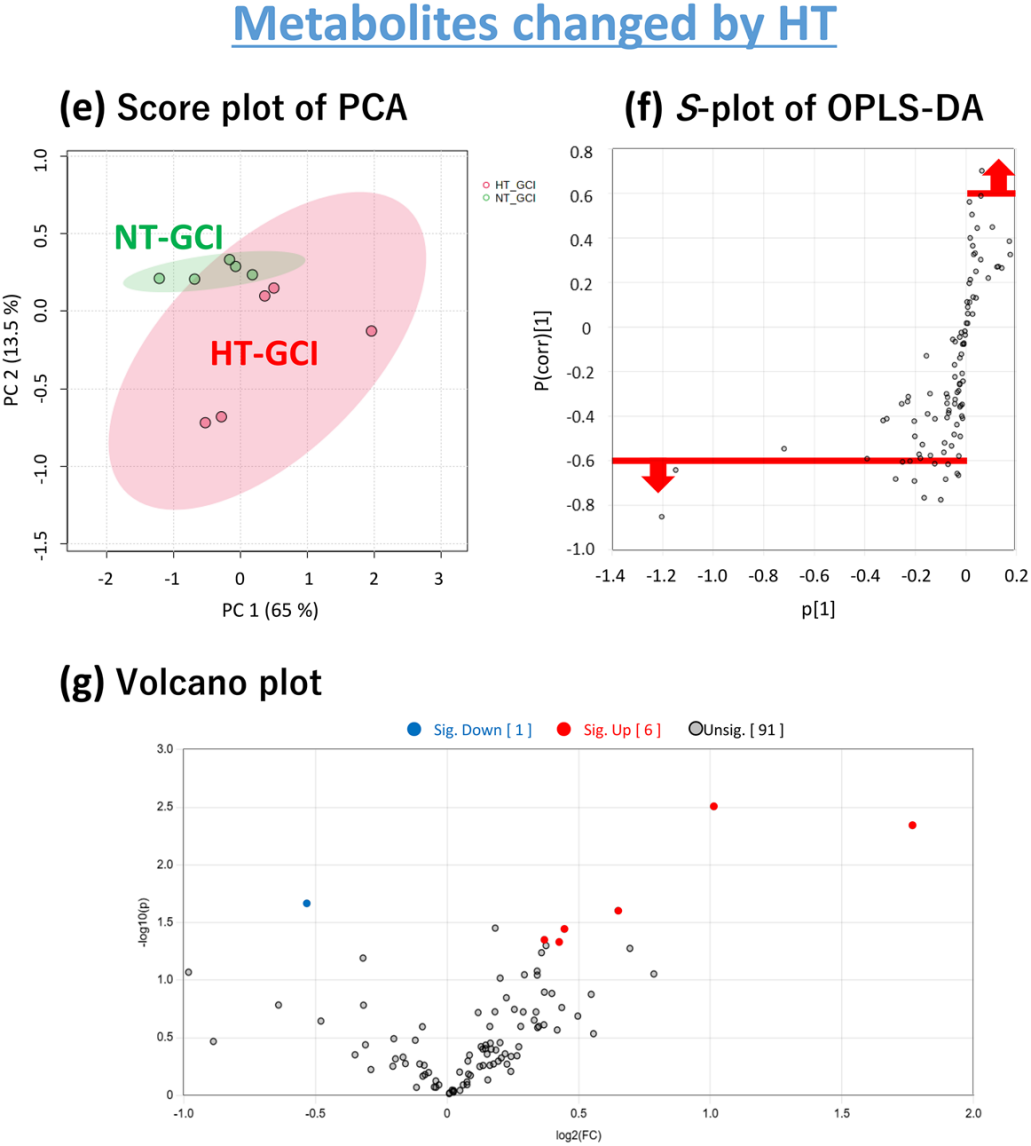
Multivariate analysis of the hippocampus 3 days after reperfusion. Score plot of PCA of all groups (**a**). Score plot of PCA of the sham-operated and GCI mice of NT group. The green and red circles indicate sham-operated and GCI mice of NT groups, respectively (**b**). *S*-plot of OPLS-DA. The vertical axis is the correlation coefficients (p (corr)^1^): the farther up or down of the axis, the more significant the difference. The horizontal axis is the covariance (p^1^): the farther to the left or right of the axis, the greater the change in level. As shown by the red line, metabolites with correlation coefficients above 0.6 are those whose levels were decreased by GCI and metabolites with correlation coefficients below − 0.6 are those whose levels were increased by GCI: these metabolites were selected (**c**). Volcano plot. The vertical axis is the − log_10_ [*p*-value], with a larger value indicating a more significant difference. The horizontal axis is the log_2_ [fold change], indicating that the farther to the left or right of the axis, the greater the amount of change. Blue circles indicate the metabolites whose levels were significantly decreased by GCI and red circles indicate those whose levels were significantly increased by GCI (**d**). Score plot of PCA. The green and red circles indicate NT and HT groups of GCI mice, respectively (**e**). As shown by the red line, metabolites with correlation coefficients above 0.6 are those whose levels were decreased by HT and metabolites with correlation coefficients below − 0.6 are those whose levels were increased by HT: these metabolites were selected (**f**). Volcano plot. Blue circles indicate the metabolites whose levels were significantly decreased by HT and red circles indicate those whose levels were significantly increased by HT (**g**).

**Figure 6:**
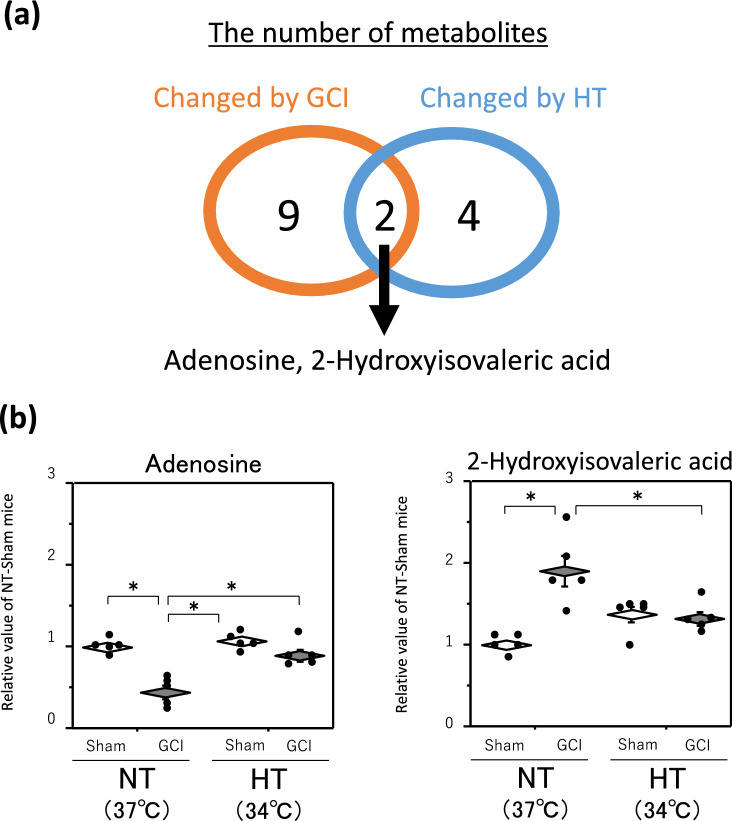

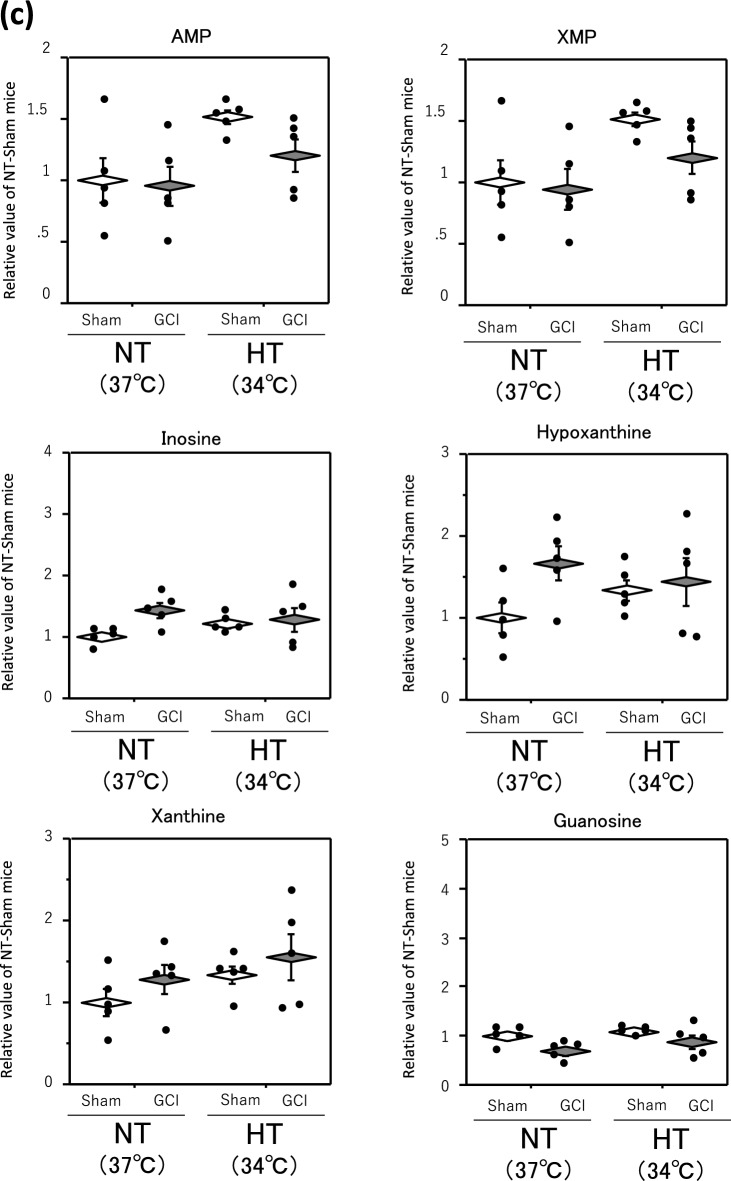
Adenosine, 2-hydroxyisovaleric acid and other purine metabolites levels in hippocampus. GCI was induced by BCCAO for 15 min under 1.5% isoflurane anesthesia in both NT and HT groups. Each control mouse (Sham) was sham-operated without BCCAO under isoflurane anesthesia. The hippocampus was removed immediately after GCI and metabolome analysis was performed as described in Materials and Methods. Venn diagram showing two shared metabolites from among metabolites whose levels were changed by GCI and HT (**a**). Adenosine and 2-hydroxyisovaleric acid levels in the hippocampus (**b**). AMP, XMP, inosine, hypoxanthine, xanthine, and guanosine levels in the hippocampus (**c**). Data are expressed as the mean and standard error of the mean for the five male mice. Statistical analyses were performed by two-way ANOVA followed by Bonferroni/Dunn post hoc test (*p < 0.0083).

We also performed a metabolome analysis 3 days after reperfusion in which HT during GCI inhibited the development of delayed neuronal death. Metabolome analysis 3 days after reperfusion was performed as described above and the results are shown in Fig. 5. Eleven metabolites whose levels were changed by GCI and 6 metabolites whose levels were changed by HT are listed in Tables 1 and 2. The metabolites changed during GCI and affected by HT could be narrowed down to two, adenosine and 2-hydroxyisovaleric acid (Fig. 6a). A detailed comparison of these two metabolites is shown in Fig. 6b. AMP, XMP and their downstream metabolites except adenosine are also shown in Fig. 6c. Adenosine levels were significantly lower in GCI mice than in sham-operated mice in the NT group, while there was no significant difference between sham-operated and GCI mice in the HT group (Fig. 6b). In contrast, AMP and XMP levels, which were reduced after GCI, returned to their basal levels in both NT and HT groups, with no significant difference between sham-operated and GCI mice (Fig. 6c). However, an increase in AMP and XMP levels by HT tended to be maintained.

## Discussion

Since Busto et al. reported in the late 1980s that lowering brain temperature in rats had a neuroprotective effect^16^, numerous animal studies have demonstrated the efficacy of therapeutic HT. Regarding the mechanism of action, many studies have focused on various molecular mechanisms involved in glutamate receptor-mediated cellular damage^17^, oxidative stress due to the production of reactive oxygen species^18^, and apoptosis in mitochondria^19^, which occur in neurons after reperfusion, including the period when neuronal death is occurring. However, although it has been shown that HT broadly suppresses these molecular mechanisms, it is difficult to distinguish whether this is due to the suppression of factors that cause neuronal death or simply a result of the suppression of the occurrence of neuronal death, and a clear mechanism has not yet been elucidated. We previously showed that the body temperature of HT mice during BCCAO does not differ from that of mice whose body temperature is maintained at 37 °C within 1 h after reperfusion^20^. Therefore, the results of the present study indicate that the difference in body temperature during GCI, when delayed neuronal death has not yet occurred, affected the occurrence of hippocampal neuronal death 3 days after reperfusion. In other words, it is highly likely that molecular changes at body temperature during GCI affected the development of delayed neuronal death in the hippocampus.

It has long been known that cerebral ischemia causes the reduction of oxygen and glucose supply, resulting in abnormal glucose metabolism, including the glycolytic system and the TCA cycle, which affects purine metabolism with the consumption of ATP, an energy substance^21^. It is also known that free fatty acids increase during cerebral ischemia by degradation of cell membrane phospholipids and that arachidonic acid metabolism is enhanced when oxygen is supplied after reperfusion. In recent years, comprehensive analysis of various metabolites including them has been carried out using mass spectrometry techniques^22^. However, the metabolic mechanisms that could explain the development of delayed neuronal death remain unclear.

We found that GCI enhances nucleic acid metabolism, particularly the purine metabolic pathway (Fig. 3). Cerebral ischemia increases ATP consumption and thus decreases the ATP levels in the brain. As a result, ATP is degraded via the pathway from AMP to adenosine and further to inosine, hypoxanthine, and xanthine^23,24^. The decrease in ATP level also causes cell injury because the influx of water and Na^+^ into the cell via Na^+^, K^+^-ATPase failure leads to the swelling of the cell and intracellular organelles^25^. On the other hand, the injury is more severe after reperfusion than during ischemia. The molecular oxygen (O_2_) supplied by reperfusion and xanthine oxidase act on hypoxanthine and xanthine to produce reactive oxygen species such as superoxide anions, hydroxyl radicals, and hydrogen peroxide, resulting in more severe injury^26^. As in the study by Rashad et al.^14^, we also demonstrated that purine metabolism was enhanced in the hippocampus after GCI (Fig. 3), consistent with these explanations, but HT during GCI had no effect on adenosine, inosine, hypoxanthine, or xanthine levels in the hippocampus (Fig. 4c). Purine metabolism also tended to be enhanced under conditions of HT during GCI (Fig. 4c). Adenosine and inosine have been reported to have neuroprotective effects via adenosine receptors against ischemic brain injury^27,28^. Therefore, these increases in levels of purine nucleotides induced by GCI may be a biological defense response to cerebral ischemia, rather than a factor in inducing delayed neuronal death in the hippocampus.

This study clearly revealed that among the metabolites whose levels were changed by GCI, only those of AMP and XMP were affected by HT (Fig. 4a and b). XMP is synthesized from inosine monophosphate (IMP) through the action of IMP dehydrogenase (IMPDH). XMP synthesis is the rate-limiting step in guanine nucleotide synthesis^29^. Because the inhibition of IMPDH suppresses the synthesis of guanine nucleotides and the proliferation of lymphocytes such as B cells and T cells, IMPDH inhibitors have been developed as anticancer and immunosuppressive agents^30^. IMPDH inhibitors have also been shown to induce apoptosis through various mechanisms^31^. Furthermore, guanosine, a guanine nucleotide, has been reported to have many protective effects against various diseases of the central nervous system, including ischemic brain damage^32–34^. Thus, an enhanced metabolism to produce guanine nucleotides can be considered to enhance their neuroprotective effects. In this study, an enhanced purine metabolism by GCI resulted in increased hippocampal guanosine levels, which were not affected by HT (Figs. 3 and 4c). Therefore, the increase in guanosine levels by GCI, as well as adenosine and inosine levels, could be regarded as a biological defense response to cerebral ischemia.

On the other hand, the occurrence of delayed neuronal death 3 days after reperfusion in the NT group could be interpreted because of the loss of neuroprotective effects due to reduced adenosine levels (Fig. 6b). However, it is not possible to determine whether changes in adenosine levels affected the development of delayed neuronal death, or its levels changed because of the occurrence of delayed neuronal death. At least adenosine levels were already changed during GCI, and changes in AMP and XMP levels by differences in body temperature during GCI may have affected adenosine levels 3 days after reperfusion. In the future, if molecules that specifically modulate AMP and XMP levels in the hippocampus during GCI are identified, those issues may be resolved by creating genetically altered mice and investigating their effects on purine metabolism in detail, using flux analysis and other techniques. 2-hydroxyisovaleric acid, which was identified along with adenosine 3 days after reperfusion, has also been reported to be detected in the urine of patients with metabolic disorders such as lactic acidosis and ketoacidosis^35^. Therefore, the significant increase in 2-hydroxyisovaleric acid in GCI mice of the NT group might reflect the result of a lack of ATP and impaired hippocampal metabolism by cerebral ischemia. However, there have been no reports of an association with cerebral ischemic pathologies, and further detailed investigation is needed.

Since HT during GCI increased AMP and XMP levels but did not affect the respective downstream metabolism of these metabolites (Fig. 4b and c), the maintenance of intracellular AMP and XMP levels may be important to prevent the development of delayed neuronal death in the hippocampus. Pannexin-1 is a nucleotide-permeable channel, and it has been shown that suppressing pannexin-1 function reduces cerebral ischemia–reperfusion injury^36^. It has also been shown that AMP, a source of ATP, is exported out of cells via pannexin-1 during apoptosis^37^. Therefore, the HT-induced increase in AMP and XMP levels may have been caused by the altered function of these channels. Further detailed studies on the expression of proteins involved in their transport into and out of the cell, as well as the enzymes involved in the synthesis and degradation of AMP and XMP, are needed in the future. In this study, in which metabolome analysis of both GCI and HT was conducted, AMP and XMP were identified as the key metabolites involved in suppressing the development of delayed neuronal death in the hippocampus (Fig. 4b). However, further investigations are needed to determine whether the maintenance of the levels of AMP and XMP in the hippocampus has a direct effect on the development of delayed neuronal death. For example, it would be necessary to clarify whether a specific increase in the levels of AMP and XMP in the hippocampus suppresses delayed neuronal death, or conversely, whether a decrease in their levels abolishes the effect of HT.

Only the levels of 10 metabolites were changed by HT, although no significant changes in the levels of other metabolites after GCI were observed (Fig. 4a and Table 2). These changes in levels of metabolites are considered to be the result of a decrease in the activity of synthesis and degradation enzymes involved in carbohydrate, amino acid, and lipid metabolisms accompanied by a decrease in body temperature. Octadecanol showed the most significant changes with HT (Table 2). It has long been known that cerebral ischemia increases the levels of free fatty acids in the brain, stearic acid and arachidonic acid^38^. Octadecanol is an alcohol derived from stearic acid and may have decreased since the level of free fatty acids was reduced by metabolic inhibition with HT. However, the role of octadecanol in the pathogenesis of cerebral ischemia is unknown and further investigation, including lipid metabolism, is required.

We performed targeted analysis of the known metabolites by GC–MS/MS. However, to elucidate the entire mechanism of delayed neuronal death in the hippocampus, it is necessary to also perform nontargeted analysis focusing on unknown molecules, as well as targeted analysis of lipids, which were not included in this study. For these approaches, the present comparative study of the effects of cerebral ischemia and HT using the BCCAO model will be very useful.

Since clinical studies have demonstrated the neuroprotective effect of HT, therapeutic HT has been developed and applied clinically at various institutions in the world. However, there has been no evidence yet of the effectiveness of HT in patients with severe head trauma or stroke. To obtain conclusive evidence, the BCCAO model of post-cardiac arrest syndrome, for which the efficacy of therapeutic HT has already been demonstrated in clinical practice, provides a promising approach to elucidating the molecular mechanism of the neuroprotective effect of HT. Furthermore, the results from the BCCAO model will greatly contribute to the elucidation of the pathophysiology of cerebral infarction analyzed using the MCAO model. We here demonstrated that HT increases AMP and XMP levels in the hippocampus, thereby preventing their decrease induced by cerebral ischemia in the BCCAO model. In the future, if the molecular mechanism of HT is elucidated based on our findings, it may lead to the development of novel neuroprotective therapies using drugs that control the molecular mechanism.

## Materials and methods

### Animals

The experimental protocol was approved by the Committee of Animal Care and Experiments of Teikyo University (22-015). All the procedures were carried out in strict compliance with the guidelines of the Committee of Animal Care and Experiments of Teikyo University. Animal studies were reported according to the ARRIVE Guidelines (Animal Research: Reporting of In Vivo Experiments). Male C57BL/6J mice were purchased from SLC Japan (Shizuoka, Japan) and acclimated for one week to the environment of animal room under a daily light (08:00–20:00)-dark cycle at room temperature in the range of 23–25 °C. The mice were housed in clear plastic cages and were fed a standard diet and water ad libitum throughout the acclimation and experimental periods. Eight- to nine-week-old mice weighing between 23 and 28 g were used in this study. After the acclimation period, mice were randomly divided in all experiments.

### Induction of GCI

GCI was induced by BCCAO with clips (Mizuho Co., Ltd., Tokyo, Japan) for 15 min under 1.5% isoflurane (FUJIFILM Wako Pure Chemical Corporation, Osaka, Japan) anesthesia in air using a face mask as described previously^15^. Cerebral blood flow after BCCAO was reduced to 15.6 ± 3.3% (n = 12) of the pre-occlusion level. Rectal temperature was monitored using a digital thermometer (Brain Science idea. Co., Ltd., Osaka, Japan) and maintained at 37 °C with a heating blanket (normothermia, NT). Hypothermia (HT) during GCI was induced by maintaining the rectal temperature at 34 °C with a heating blanket. Each control mouse underwent a sham operation without BCCAO under isoflurane anesthesia for 15 min. All surgeries were performed between 9am and 1 pm. No mice died during surgery or after GCI.

### Analysis of delayed neuronal death in hippocampus

A total of 48 mice were used to assess the delayed neuronal death in the hippocampus and were included in the study. The nucleosome level in the hippocampus after GCI was quantitatively assayed using a cell death detection enzyme-linked immunosorbent assay (ELISA) kit (Roche Diagnostics, Indianapolis, IN, USA), as described previously^15^. The hippocampus was dissected 1, 3, and 7 days after GCI (Fig. 1a) and homogenized using a disposable homogenizer (Nippi, Tokyo, Japan) in the incubation buffer (10 μL/mg tissue) for the ELISA kit. The homogenate was centrifuged at 20,000×*g* for 10 min after incubation for 30 min at room temperature, and the supernatant diluted fivefold was used for the assay.

### Sample preparation for metabolome quantification

A total of 40 mice were used for metabolome analysis and were included in the study. The hippocampus was removed immediately after the end of GCI (Fig. 1a), frozen in liquid nitrogen, and stored at − 80 °C until use for metabolome analysis. Then the hippocampus was homogenized with 800 µL of methanol (FUJIFILM Wako Pure Chemical Corporation) and 5 × 2.8 mm silica beads using Percellys24 and Cryolys (Bertin Technologies, Montigny-le-Bretonneux, France). Homogenization was conducted in three cycles of 6800 rpm for 15 s at intervals of 30 s under cold nitrogen flow using liquid nitrogen. 20 µLof homogenates was diluted with 80 µL of methanol. Then, 100 µL of 1 µg/mL glutamic acid ^13^C_5_^15^N_1_ (IS) (Toronto Research Chemicals, Ontario, Canada) in distilled water (DW, FUJIFILM Wako Pure Chemical Corporation) and 200 µL of methanol:CHCl_3_ (FUJIFILM Wako Pure Chemical Corporation) (1:1) solution were added into the diluted specimens. Extraction was performed using Thermo-Shaker TS-100C and SC-24NC (BIOSAN Medical-Biological Research and Technologies, Riga, Latvia) at 1400 rpm for 15 min at 4 °C. The sample was centrifuged at 16,000×*g* for 5 min at 4 °C, and 200 µLof the supernatant was transferred into a new 1.5 mL tube containing 200 µL of DW. The sample was extracted and centrifuged under the same conditions mentioned above. 300 µL of the supernatant was transferred into a new tube, and centrifugal concentration was performed for 10 min using CentriVap Mobile Systems (Labconco Corporation, MO, USA). The sample was frozen with liquid nitrogen and freeze-dried using an EYELA FDM-1000 freeze dryer (Tokyo Rika Kiki Co., Ltd. Tokyo, Japan) overnight. The next day, 40 µL of 20 mg/mL methoxyamine hydrochloride (for GC derivatization, Merck KGaA, Darmstadt, Germany) in pyridine (FUJIFILM Wako Pure Chemical Corporation) was added to the sample. Then the sample was incubated at 1400 rpm for 90 min at 30 °C. After spinning down, 20 µL of *N*-methyl-*N*-(trimethylsilyl)trifluoroacetamide (Merk KgaA) was added to the sample and incubated at 1,400 rpm for 30 min at 37 °C. 50 µL of the sample was transferred to a glass insert containing a glass vial, and a 1 µL aliquot was injected into a gas chromatograph-tandem mass spectrometer.

### Analytical conditions for GC–MS/MS

GCMSTQ8040 and GCMS solutions (Shimadzu, Kyoto, Japan) were employed as a gas chromatography-tandem mass spectrometer system, and a DB-5 capillary column (30 m × 0.25 mm id, df = 1.00 μm) (Agilent Technologies, Santa Clara, CA, USA) was used for separation. The column oven was programmed as follows the initial temperature was set at 100 °C and held for 4 min, then the temperature was linearly increased to 320 °C at 10 °C/min and held for 11 min at 320 °C. High-purity helium gas was employed as the carrier gas, and the flow rate was 1.1 mL/min. Electroionization was conducted at 70 eV. One µL of an aliquot of the sample was injected into the GC–MS/MS system in the splitless mode. Selective reaction monitoring (SRM) was conducted to quantify and identify the metabolites. SRM transitions and retention times of quantified metabolites in this study are shown in Supplemental Table 1. Each metabolite was quantified using the area under the curve of a mass chromatogram for quantified ions, and the metabolite quantity was normalized by the area of glutamic acid ^13^C_5_^15^N and hippocampus weight.

### Statistical analyses

Comparisons of nucleosome level were performed by two-way ANOVA followed by Bonferroni/Dunn post hoc test. The data obtained were analyzed using MetaboAnalyst 5.0 software. Metabolites showing statistically significant differences in levels were identified by the raw *p*-value detected by the software. When the *p*-value was lower than 0.05, the difference was considered significant, and the candidate metabolites were narrowed down^39^. For individual metabolites, statistical analyses were performed by two-way ANOVA followed by Bonferroni/Dunn post hoc test using Stat View 5.0 software. The significance level was set at 5%. There was no interaction between the effects of GCI and body temperature, except for adenosine and 2-hydroxyisovaleric acid levels 3 days after reperfusion.

### Supplementary Information

Supplementary Information.

## Data Availability

The datasets obtained in this study are available from the corresponding author on reasonable request.

## References

[1] Volpe BT, Petito CK (1985). Dementia with bilateral medial temporal lobe ischemia. Neurology.

[2] Petito CK, Feldmann E, Pulsinelli WA, Plum F (1987). Delayed hippocampal damage in humans following cardiorespiratory arrest. Neurology.

[3] Bernard SA (2002). Treatment of comatose survivors of out-of-hospital cardiac arrest with induced hypothermia. N. Engl. J. Med..

[4] Liu L, Yenari MA (2009). Clinical application of therapeutic hypothermia in stroke. Neurol. Res..

[5] Kirino T (1982). Delayed neuronal death in the gerbil hippocampus following ischemia. Brain Res..

[6] Kawase M (1999). Exacerbation of delayed cell injury after transient global ischemia in mutant mice with CuZn superoxide dismutase deficiency. Stroke.

[7] Sun HS (2009). Suppression of hippocampal TRPM7 protein prevents delayed neuronal death in brain ischemia. Nat. Neurosci..

[8] Tajiri S (2004). Ischemia-induced neuronal cell death is mediated by the endoplasmic reticulum stress pathway involving CHOP. Cell Death Differ..

[9] Doshi M, Kuwatori Y, Ishii Y, Sasahara M, Hirashima Y (2009). Hypothermia during ischemia protects against neuronal death but not acute brain edema following transient forebrain ischemia in mice. Biol. Pharm. Bull..

[10] Soga T (2006). Differential metabolomics reveals ophthalmic acid as an oxidative stress biomarker indicating hepatic glutathione consumption. J. Biol. Chem..

[11] Yachida S (2019). Metagenomic and metabolomic analyses reveal distinct stage-specific phenotypes of the gut microbiota in colorectal cancer. Nat. Med..

[12] Miura D (2010). Ultrahighly sensitive in situ metabolomic imaging for visualizing spatiotemporal metabolic behaviors. Anal. Chem..

[13] Irie M, Fujimura Y, Yamato M, Miura D, Wariishi H (2014). Integrated MALDI-MS imaging and LC-MS techniques for visualizing spatiotemporal metabolomic dynamics in a rat stroke model. Metabolomics.

[14] Rashad S (2020). Metabolic basis of neuronal vulnerability to ischemia; an in vivo untargeted metabolomics approach. Sci. Rep..

[15] Doshi M, Watanabe S, Natori Y, Hosoyamada M, Hirashima-Akae Y (2021). Triiodothyronine aggravates global cerebral ischemia-reperfusion injury in mice. Biol. Pharm. Bull..

[16] Busto R (1987). Small differences in intraischemic brain temperature critically determine the extent of ischemic neuronal injury. J. Cereb. Blood Flow Metab..

[17] Kataoka K, Yanase H (1998). Mild hypothermia-a revived countermeasure against ischemic neuronal damages. Neurosci. Res..

[18] Yu H (2020). Protective effects of combined treatment with mild hypothermia and edaravone against cerebral ischemia/reperfusion injury via oxidative stress and Nrf2 pathway regulation. Int. J. Oncol..

[19] Zhao H, Yenari MA, Cheng D, Sapolsky RM, Steinberg GK (2005). Biphasic cytochrome c release after transient global ischemia and its inhibition by hypothermia. J. Cereb. Blood Flow Metab..

[20] Doshi M, Higuchi E, Hirashima Y (2011). Hypothermia after reperfusion suppresses aggravation of acute brain edema following transient forebrain ischemia in mice. J. Health Sci..

[21] Siesjö BK (1984). Cerebral circulation and metabolism. J. Neurosurg..

[22] Shin TH, Lee DY, Basith S, Manavalan B, Paik MJ, Rybinnik I, Mouradian MM, Ahn JH, Lee G (2020). Metabolome changes in cerebral ischemia. Cells..

[23] Kanemitsu H (1988). Xanthine and uric acid levels in rat brain following focal ischemia. J. Neurochem..

[24] Nihei H, Kanemitsu H, Tamura A, Oka H, Sano K (1989). Cerebral uric acid, xanthine, and hypoxanthine after ischemia: the effect of allopurinol. Neurosurgery..

[25] Kempski O (2001). Cerebral edema. Semin. Nephrol..

[26] Chan PH (1996). Role of oxidants in ischemic brain damage. Stroke.

[27] Seydyousefi M (2019). Exogenous adenosine facilitates neuroprotection and functional recovery following cerebral ischemia in rats. Brain Res. Bull..

[28] Shen H, Chen GJ, Harvey BK, Bickford PC, Wang Y (2005). Inosine reduces ischemic brain injury in rats. Stroke.

[29] Zimmermann AG, Gu JJ, Laliberte J, Mitchell BS (1998). Inosine-5’-monophosphate dehydrogenase: regulation of expression and role in cellular proliferation and T lymphocyte activation. Prog. Nucleic Acid Res. Mol. Biol..

[30] Jain J, Almquist SJ, Shlyakhter D, Harding MW (2001). VX-497: a novel, selective IMPDH inhibitor and immunosuppressive agent. J. Pharm. Sci..

[31] Camici M, Garcia-Gil M, Pesi R, Allegrini S, Tozzi MG (2019). Purine-metabolising enzymes and apoptosis in cancer. Cancers..

[32] Hansel G (2014). The potential therapeutic effect of guanosine after cortical focal ischemia in rats. PLoS One..

[33] Hansel G (2015). Guanosine protects against cortical focal ischemia. Involve. Inflamm. Response. Mol. Neurobiol..

[34] Ramos DB (2016). Intranasal guanosine administration presents a wide therapeutic time window to reduce brain damage induced by permanent ischemia in rats. Purinergic Signal..

[35] Landaas S, Jakobs C (1977). The occurrence of 2-hydroxyisovaleric acid in patients with lactic acidosis and ketoacidosis. Clin. Chim. Acta..

[36] Bargiotas P (2011). Pannexins in ischemia-induced neurodegeneration. Proc. Natl. Acad. Sci. U S A.

[37] Imamura H (2020). Single-cell dynamics of pannexin-1-facilitated programmed ATP loss during apoptosis. Elife.

[38] Yoshida S, Inoh S, Asano T, Sano K, Kubota M, Shimazaki H, Ueta N (1980). Effect of transient ischemia on free fatty acids and phospholipids in the gerbil brain. Lipid peroxidation as a possible cause of postischemic injury. J. Neurosurg..

[39] Kikuchi K (2019). Gut microbiome-derived phenyl sulfate contributes to albuminuria in diabetic kidney disease. Nat. Commun..

