# Supplementary material for: Hypothermia increases adenosine monophosphate and xanthosine monophosphate levels in the mouse hippocampus, preventing their reduction by global cerebral ischemia
Source: Sci Rep. 2024 Feb 7;14:3187. doi: 10.1038/s41598-024-53530-1 (PMC10850059; doi:10.1038/s41598-024-53530-1)
Supplement: Supplementary file 1 — Supplementary Information. [file 41598_2024_53530_MOESM1_ESM.pdf]

Supplemental Table 1.

Selected Reaction Monitoring (SRM) transitions and retention times of quantified metabolites

metabolite name	derivative	Retention Time (min)	quantify ion (m/z)	qualify ion (m/z)
Pyruvic acid	meto-TMS	8.083	174.00>74.00	174.00>89.00
Glycolic acid	2TMS	8.611	177.10>147.10	177.10>73.00
Caproic acid	TMS	8.631	173.10>75.00	117.10>75.00
Glycine	2TMS	9.457	176.10>147.10	204.10>147.10
Oxalic acid	2TMS	9.583	190.10>147.10	219.10>147.10
2-Hydroxyisovaleric acid	2TMS	10.315	219.20>147.10	247.20>147.10
3-Hydroxyisovaleric acid	2TMS	11.081	131.10>73.00	247.10>73.00
Valine	2TMS	11.227	144.10>73.00	218.10>147.10
Benzoic acid	TMS	11.793	179.10>105.10	179.10>77.00
Octanoic acid	TMS	11.890	201.10>75.00	117.10>75.00
2-Aminoethanol	3TMS	12.100	174.10>73.00	174.10>86.10
Leucine	2TMS	12.100	158.10>73.00	158.10>102.10
Isoleucine	2TMS	12.472	158.20>73.00	218.10>73.00
4-Aminobutyric acid	2TMS	12.534	115.10>100.10	232.20>147.10
Maleic acid	2TMS	12.564	245.10>147.10	245.10>73.00
Proline	2TMS	12.608	142.10>73.00	216.10>147.10
Methylsuccinic acid	2TMS	12.860	261.20>147.10	217.20>55.00
Glyceric acid	3TMS	12.948	189.10>73.00	292.10>73.00

Fumaric acid	2TMS	13.096	245.00>147.10	143.10>75.00
Uracil	2TMS	13.141	241.10>147.10	241.10>99.00
Nonanoic acid	TMS	13.348	215.10>75.00	215.10>131.10
Threonine	3TMS	13.836	218.10>73.00	291.00>101.10
Glutaric acid	2TMS	13.954	261.00>147.10	233.10>147.10
3-Aminopropanoic acid	3TMS	14.475	248.10>147.10	290.10>248.20
Decanoic acid	TMS	14.748	117.10>75.00	229.20>75.10
Malic acid	3TMS	15.158	335.00>147.10	307.00>147.10
Niacinamide	TMS	15.267	179.10>75.00	179.10>136.10
Dihydrouracil	2TMS	15.450	243.10>147.10	243.10>201.10
Methionine	2TMS	15.731	250.10>147.10	293.10>232.10
Cysteine	3TMS	16.164	220.10>73.00	218.10>73.00
2-Hydroxyglutaric acid	3TMS	16.278	247.20>129.10	247.20>73.00
Creatinine	3TMS	16.291	329.20>115.10	329.20>143.10
Ornithine	3TMS	16.826	142.10>73.00	142.10>58.10
Glutamic acid-13C5	3TMS	16.861	251.10>132.00	235.00>147.00
Hypotaurine	3TMS	16.885	188.10>73.00	188.10>100.10
Cadaverine	3TMS	16.957	174.10>73.00	174.10>86.00
Phenylalanine	2TMS	17.172	218.10>73.00	192.10>73.00
Lauric acid	TMS	17.221	257.10>75.00	132.10>117.10
2-Ketoadipic acid	2TMS	17.340	258.10>73.00	302.10>73.00
Asparagine	3TMS	17.543	188.10>73.00	231.10>132.10

N-Acetylaspartic acid	3TMS	17.591	274.10>73.00	274.10>184.10
Ribose	meto 4TMS	17.651	307.10>73.00	307.10>217.10
1,6-Anhydroglucose	3TMS	18.079	204.10>73.00	204.10>189.10
Aconitic acid	3TMS	18.293	285.10>147.10	375.10>147.10
2-Deoxy-glucose	4TMS	18.327	204.10>73.00	204.10>189.10
Glutamine	4TMS	18.373	203.10>147.10	227.10>73.00
Putrescine	4TMS	18.531	174.10>73.00	174.10>86.00
Dihydroxyacetone phosphate	meto 3TMS	18.531	315.10>73.00	315.10>299.00
Glycerol 3-phosphate	4TMS	18.576	357.10>73.00	357.10>341.10
O-Phosphoethanolamine	4TMS	18.960	328.10>298.10	414.10>299.10
3-Phosphoglyceric acid	4TMS	19.110	387.20>73.00	459.10>299.10
2-Aminopimelic acid	3TMS	19.163	274.20>73.00	274.20>184.20
Isocitric acid	4TMS	19.185	245.10>73.00	245.10>83.00
Hypoxanthine	2TMS	19.249	265.10>73.00	280.10>265.10
Citric acid	4TMS	19.249	347.10>147.10	347.10>257.10
Dopamine	3TMS	19.399	102.10>73.00	102.10>58.00
Psicose	meto 5TMS	19.892	307.10>73.00	307.10>217.10
Tagatose	meto 5TMS	19.903	307.10>217.10	307.10>103.10
Sorbose	meto 5TMS	19.957	307.10>217.10	307.10>73.00
Adenine	2TMS	19.989	264.10>73.00	279.20>264.10
Glucose	meto 5TMS	20.255	319.10>73.00	319.10>129.10
Lysine	4TMS	20.378	317.20>156.20	317.20>73.00

Tyramine	3TMS	20.439	174.10>73.00	174.10>86.00
Tyrosine	3TMS	20.612	218.10>73.00	218.10>100.10
Sorbitol	6TMS	20.734	319.10>73.00	319.10>129.10
Pantothenic acid	3TMS	21.157	291.20>201.10	291.20>159.10
Palmitoleic acid	TMS	21.313	311.10>75.00	311.10>131.10
Xanthine	3TMS	21.410	353.10>147.10	368.10>353.10
Ribose 5-phosphate	meto 5TMS	22.302	315.10>73.00	315.10>299.10
Octadecanol	TMS	22.506	327.30>75.00	103.10>73.00
Norepinephrine	5TMS	22.878	174.10>73.00	174.10>86.00
Oleic acid	TMS	23.139	339.30>75.00	199.10>109.10
Elaidic acid	TMS	23.166	339.20>75.00	339.20>131.10
Tryptophan	3TMS	23.397	202.10>73.00	291.10>101.10
Arachidonic acid	TMS	24.577	117.10>75.00	93.10>77.00
2'-Deoxyuridine	3TMS	25.221	241.10>99.00	241.10>147.10
Uridine	4TMS	25.327	224.10>73.00	224.10>209.10
Inositol phosphate	7TMS	25.392	318.20>73.00	387.10>73.00
5-Methoxytryptamine	2TMS	25.425	174.20>73.00	174.20>86.00
Inosine	4TMS	26.324	259.10>73.00	281.10>73.00
Adenosine	4TMS	26.890	230.10>73.00	245.10>73.00
Sucrose	8TMS	27.105	361.10>73.00	361.10>169.10
Guanosine	5TMS	28.137	324.10>264.10	368.20>73.00
Xanthosine monophosphate	6TMS	32.160	315.20>73.00	315.20>299.10

Adenosine monophosphate	5TMS	32.195	382.10>125.10	382.10>140.10
